# Supplementary material for: Differential Regulation of Nav1.1 and SCN1A Disease Mutant Sodium Current Properties by Fibroblast Growth Factor Homologous Factors
Source: Cells. 2025 Feb 15;14(4):291. doi: 10.3390/cells14040291 (PMC11853998; doi:10.3390/cells14040291)
Supplement: Supplementary file 1 [file cells-14-00291-s001.zip › cells-3190660-supplementary.pdf]

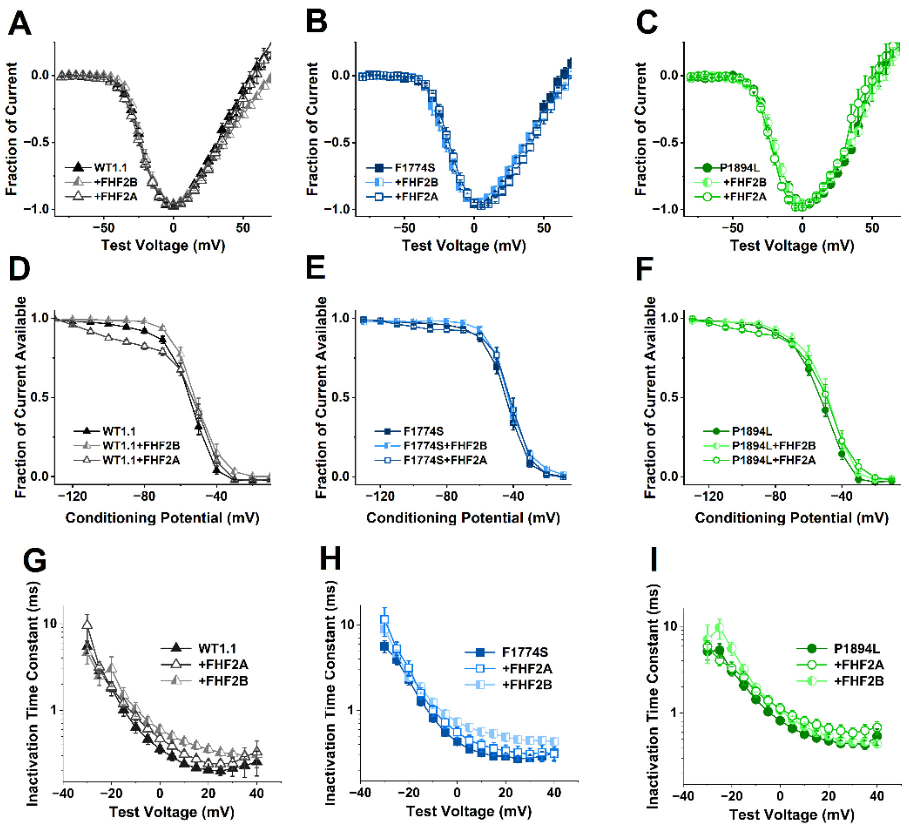


Figure S1. Limited impact of FHF2A and FHF2B on basic biophysical properties of WT and mutant Nav1.1 channels. (A-C) The normalized current–voltage (*I*–*V*) properties were assessed using depolarizing step pulses. Cells were held at −100 mV. The currents were elicited by 50 ms test depolarizations to various potentials from −80 to +65 mV stepped in increments of 5 mV. The peak current evoked by each pulse, normalized to the maximum peak current, is plotted versus the test voltage. There was no significant difference in the voltage-dependence of activation for WT (A), F1774S (B) and P1894L (C) with either FHF2A or FHF2B co-expression. (D-F) Comparison of steady-state inactivation for WT (D), F1774S (E) and P1894L (G) channels under control conditions or with FHF2A or FHF2B. (G-I) Rate of transient current decay for for WT (G), F1774S (H) and P1894L (I) channels under control conditions or with FHF2A or FHF2B. FHF2B slightly slowed the rate of decay for WT and F1774S channels at voltages around +20 mV, but not at more negative voltages.

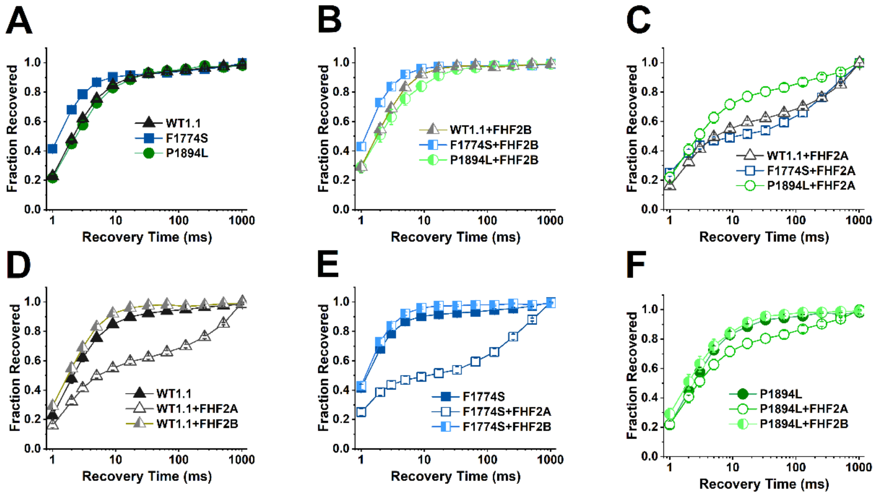


**Figure S2.** The F1774S mutation and FHF2A differentially impact recovery from inactivation. Recovery from inactivation time course is shown for HEK293 cells transiently expressing human WT, F1774S and P1894L under control conditions (A), with FHF2B (B) and with FHF2A (C). (D) Recovery from inactivation of WT channels under all three conditions. (E) Recovery from inactivation of F1774S channels under all three conditions. (F) Recovery from inactivation of P1894L channels under all three conditions.

## Table S1: Fraction Available Pulse 5 - Descriptive Statistics

| | N Analysis | Mean | Standard Deviation | SE of Mean |
|---|---|---|---|---|
| WT1.1 (WT) | 33 | 0.91262 | 0.06247 | 0.01087 |
| WT1.1+FHF2B (WT2B) | 21 | 0.9715 | 0.02011 | 0.00439 |
| WT1.1+FHF2A (WT2A) | 26 | 0.35284 | 0.10513 | 0.02062 |
| F1774S (FS) | 23 | 0.87091 | 0.13144 | 0.02741 |
| F1774S+FHF2B (FS2B) | 22 | 0.99719 | 0.0304 | 0.00648 |
| F1774S+FHF2A (FS2A) | 20 | 0.26481 | 0.08677 | 0.0194 |
| P1894L (PL) | 26 | 0.95996 | 0.054 | 0.01059 |
| P1894L +FHF2B (PL2B) | 22 | 0.96758 | 0.08615 | 0.01837 |
| P1894L +FHF2A (PL2A) | 22 | 0.74146 | 0.14808 | 0.03157 |

## Table S2: LTI Pulse 5: Means Comparisons Post-Hoc Tukey Test

| | MeanDiff | SEM | q Value | Prob | Alpha | Sig | LCL | UCL |
|---|---|---|---|---|---|---|---|---|
| WT2B WT | 0.05888 | 0.02491 | 3.34192 | 0.31011 | 0.05 | 0 | -0.01923 | 0.13699 |
| WT2A WT | -0.55978 | 0.02341 | 33.82415 | <0.0001 | 0.05 | 1 | -0.63316 | -0.48641 |
| WT2A WT2B | -0.61866 | 0.02619 | 33.41089 | <0.0001 | 0.05 | 1 | -0.70076 | -0.53656 |
| FS WT | -0.04171 | 0.02424 | 2.43299 | 0.73338 | 0.05 | 0 | -0.11772 | 0.0343 |
| FS WT2B | -0.10058 | 0.02694 | 5.2804 | 0.00738 | 0.05 | 1 | -0.18504 | -0.01613 |
| FS WT2A | 0.51808 | 0.02555 | 28.67704 | <0.0001 | 0.05 | 1 | 0.43798 | 0.59817 |
| FS2B WT | 0.08457 | 0.02457 | 4.8686 | 0.01965 | 0.05 | 1 | 0.00755 | 0.16159 |
| FS2B WT2B | 0.0257 | 0.02723 | 1.3346 | 0.9901 | 0.05 | 0 | -0.05967 | 0.11106 |
| FS2B WT2A | 0.64436 | 0.02586 | 35.24457 | <0.0001 | 0.05 | 1 | 0.5633 | 0.72542 |
| FS2B FS | 0.12628 | 0.02662 | 6.70958 | 1.34072E-4 | 0.05 | 1 | 0.04283 | 0.20973 |
| FS2A WT | -0.64782 | 0.02529 | 36.22213 | <0.0001 | 0.05 | 1 | -0.72711 | -0.56852 |
| FS2A WT2B | -0.70669 | 0.02789 | 35.83861 | <0.0001 | 0.05 | 1 | -0.79412 | -0.61926 |
| FS2A WT2A | -0.08803 | 0.02655 | 4.68974 | 0.02923 | 0.05 | 1 | -0.17126 | -0.00481 |
| FS2A FS | -0.60611 | 0.02729 | 31.41106 | <0.0001 | 0.05 | 1 | -0.69166 | -0.52055 |
| FS2A FS2B | -0.73239 | 0.02758 | 37.56051 | <0.0001 | 0.05 | 1 | -0.81884 | -0.64594 |
| PL WT | 0.04734 | 0.02341 | 2.86017 | 0.52958 | 0.05 | 0 | -0.02604 | 0.12071 |
| PL WT2B | -0.01154 | 0.02619 | 0.62325 | 0.99996 | 0.05 | 0 | -0.09364 | 0.07056 |
| PL WT2A | 0.60712 | 0.02475 | 34.68442 | <0.0001 | 0.05 | 1 | 0.52951 | 0.68473 |
| PL FS | 0.08904 | 0.02555 | 4.92885 | 0.01712 | 0.05 | 1 | 0.00895 | 0.16914 |
| PL FS2B | -0.03724 | 0.02586 | 2.03677 | 0.88085 | 0.05 | 0 | -0.1183 | 0.04382 |
| PL FS2A | 0.69515 | 0.02655 | 37.03313 | <0.0001 | 0.05 | 1 | 0.61193 | 0.77838 |
| PL2B WT | 0.05496 | 0.02457 | 3.16364 | 0.38589 | 0.05 | 0 | -0.02206 | 0.13197 |
| PL2B WT2B | -0.00392 | 0.02723 | 0.20361 | 1 | 0.05 | 0 | -0.08929 | 0.08145 |
| PL2B WT2A | 0.61474 | 0.02586 | 33.62461 | <0.0001 | 0.05 | 1 | 0.53368 | 0.6958 |
| PL2B FS | 0.09666 | 0.02662 | 5.13598 | 0.01051 | 0.05 | 1 | 0.01322 | 0.18011 |
| PL2B FS2B | -0.02962 | 0.02691 | 1.55641 | 0.97363 | 0.05 | 0 | -0.11399 | 0.05475 |
| *PL2B FS2A | 0.70277 | 0.02758 | 36.04161 | 1.5163E-4 | 0.05 | 1 | 0.61632 | 0.78922 |
| PL2B PL | 0.00762 | 0.02586 | 0.41681 | 1 | 0.05 | 0 | -0.07344 | 0.08868 |
| PL2A WT | -0.17116 | 0.02457 | 9.85318 | <0.0001 | 0.05 | 1 | -0.24818 | -0.09414 |
| PL2A WT2B | -0.23004 | 0.02723 | 11.9473 | <0.0001 | 0.05 | 1 | -0.3154 | -0.14467 |
| PL2A WT2A | 0.38862 | 0.02586 | 21.25673 | <0.0001 | 0.05 | 1 | 0.30757 | 0.46968 |
| PL2A FS | -0.12945 | 0.02662 | 6.878 | <0.0001 | 0.05 | 1 | -0.2129 | -0.046 |
| PL2A FS2B | -0.25573 | 0.02691 | 13.43908 | <0.0001 | 0.05 | 1 | -0.3401 | -0.17136 |
| PL2A FS2A | 0.47666 | 0.02758 | 24.44531 | <0.0001 | 0.05 | 1 | 0.3902 | 0.56311 |
| PL2A PL | -0.21849 | 0.02586 | 11.95107 | <0.0001 | 0.05 | 1 | -0.29955 | -0.13744 |
| PL2A PL2B | -0.22612 | 0.02691 | 11.88267 | <0.0001 | 0.05 | 1 | -0.31048 | -0.14175 |

Sig equals 1 indicates that the difference of the means is significant at the 0.05 level. Sig equals 0 indicates that the difference of the means is not significant at the 0.05 level.
